# Experimental dataset for optimising the freight rail operations

**DOI:** 10.1016/j.dib.2016.09.015

**Published:** 2016-09-21

**Authors:** Mahmoud Masoud, Erhan Kozan, Geoff Kent, Shi Qiang Liu

**Affiliations:** a Science and Engineering Faculty, Queensland University of Technology, 2 George St, GPO Box 2434, Brisbane, QLD 4001, Australia; b School of Transportation and Logistics, Southwest Jiaotong University, Chengdu City 6117563, Sichuan Province, China

**Keywords:** Fright Rail Systems, Train Scheduling, Metaheuristic, Constraint Programming

## Abstract

The freight rail systems have an essential role to play in transporting the commodities between the delivery and collection points at different locations such as farms, factories and mills. The fright transport system uses a daily schedule of train runs to meet the needs of both the harvesters and the mills (An Integrated Approach to Optimise Cane Rail Operations (M. Masoud, E. Kozan, G. Kent, Liu, Shi Qiang, 2016b) [1]). Producing an efficient daily schedule to optimise the rail operations requires integration of the main elements of harvesting, transporting and milling in the value chain of the Australian agriculture industry. The data utilised in this research involve four main tables: sidings, harvesters, sectional rail network and trains. The utilised data were collected from Australian sugar mills as a real application. Operations Research techniques such as metaheuristic and constraint programming are used to produce the optimised solutions in an analytical way.

## Specifications Table

**Table:** 

Subject area	Operations Research
More specific subject area	Rail Systems Optimisation
Type of data	Table, graph, figure
How data was acquired	From mills and farm locations
Data format	Filtered, analysed
Experimental factors	Data had been customised to remove any mismatching with real life application such as siding capacity, daily allotment,
Experimental features	A near optimal scheduler for trains was produced using a real sector of Australian rail network.
Data source location	Queensland University of Technology, Brisbane, Australia
Data accessibility	Data is within this article

## Value of Data

• The main aim of the presented data is to develop mathematical models of the freight rail systems and help in producing effective solutions in a reasonable CPU time.
• In this research, minimising the makespan is proposed as a main criterion to optimise the freight rail systems using the introduced data. The results in this research can be used to compare the performance of the proposed mathematical methods in optimising complex systems such as rail systems in many prospective studies.
• The data of the produced schedules of the train runs can be used for many different types of the freight systems such as the sugarcane or coal rail systems [5]. The data describe the daily trips of each train to deliver the empty bins at different locations called sidings and collect the full bins from these sidings for delivery to the mills or the factories.

## 1 Data

**Fig. 1:**
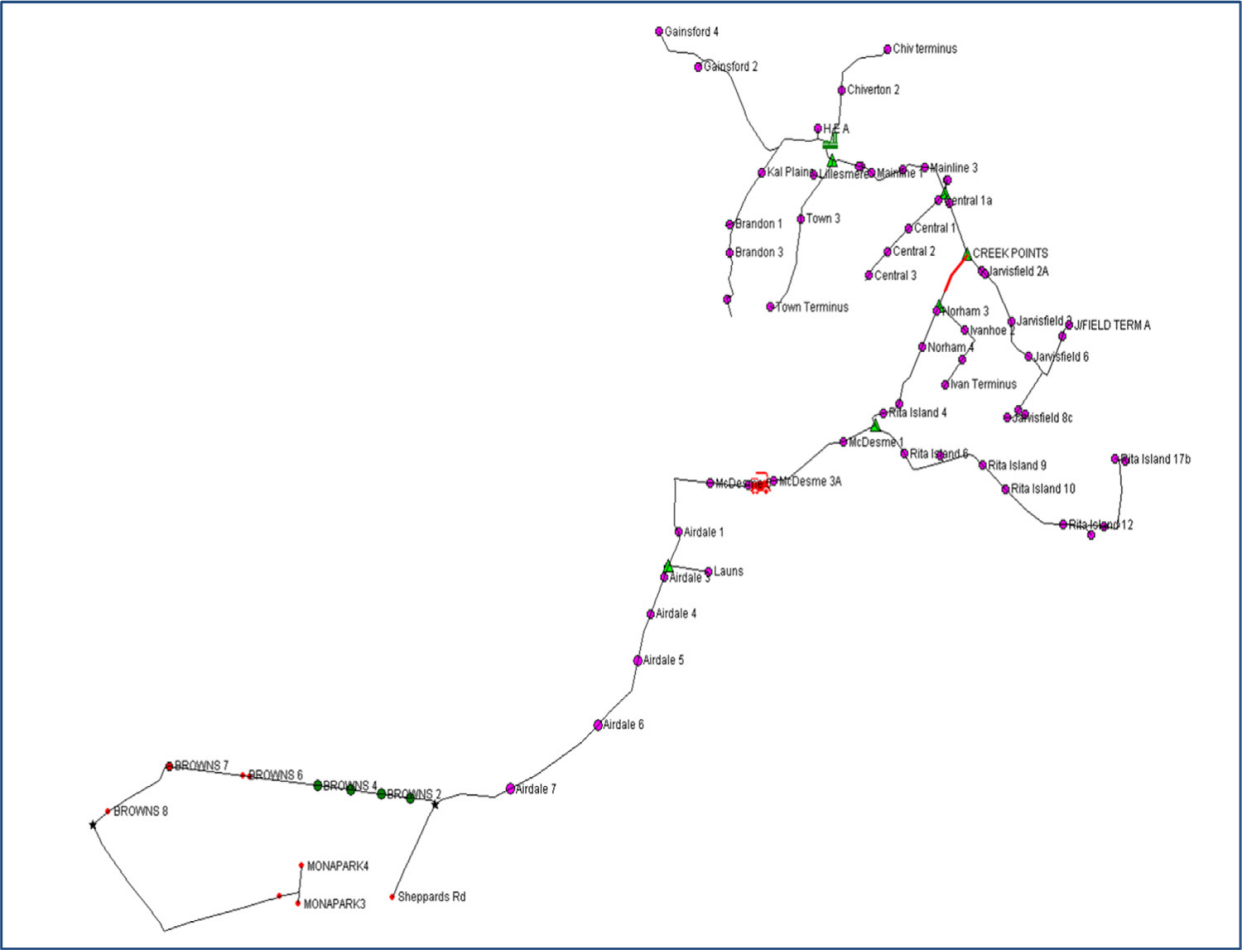
A sector of the rail transport system of Kalamia’s mill.

**Fig. 2:**
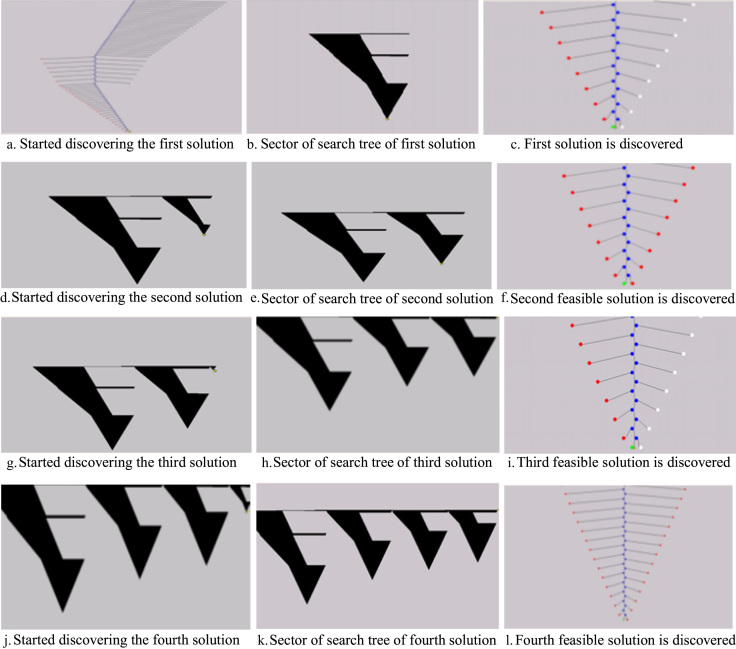
Search tree of the stages for discovering four feasible solutions using DFS. a. Started discovering the first solution b. Sector of search tree of first solution c. First solution is discovered. d. Started discovering the second solution e. Sector of search tree of second solution f. Second feasible solution is discovered. g. Started discovering the third solution h. Sector of search tree of third solution i. Third feasible solution is discovered. j. Started discovering the fourth solution k. Sector of search tree of fourth solution l. Fourth feasible solution is discovered.

**Fig. 3:**
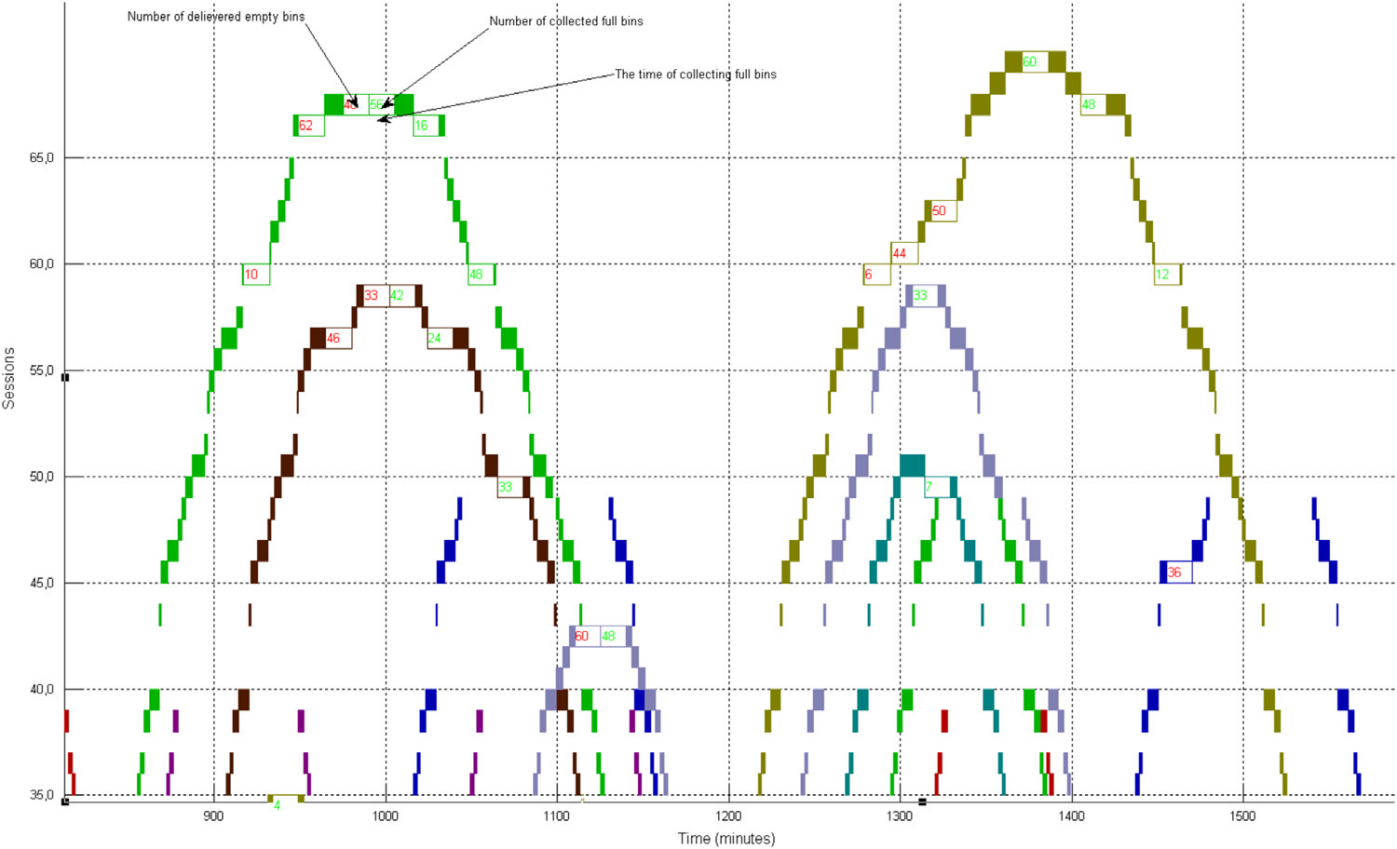
A Gantt chart showing delivered and collected bins and shunting times.

Based on the feedback from our industry partners, the data utilised in this research are created in four main tables: Sidings (Table 1), Trains (Table 2), Harvesters (Table 3) and Rail Network (Table 4). In addition, three figures are presented to show the main steps of the proposed solutions: Kalamia’s mill with the main original map (Fig. 1), the main steps to produce the final solution (Fig. 2), and the daily trips of each train in the system (Fig. 3).

## 2 Experimental design, materials and methods

A case study was examined to validate the constraint programming models and metaheuristic techniques. Fig. 1 shows a sector of the transport system of Townsville’s mill in Queensland, Australia. Many train runs are generated where each run start at one mill and finishes at the same mill after visiting many different siding locations. The number of trains was selected to implement different runs requiring a fewer number of trains. Kalamia’s mill has 58 sidings located in 9 segments but not all of them work on the same day. Approximately 14 trains can be used to construct the train trips that deliver empty bins to sidings at farms and collect full bins from farms top sidings. The data table of sectional rail network was constructed to describe the rail section length between different sidings.

Constraint programming (CP) is one of solution techniques to find a near optimal scheduler for the sugarcane rail systems. The proposed mathematical model considers the siding and train capacity constraints, daily allotment constraints of each harvester, train passing constraints where each train cannot occupy more than one rail section at a time or two trains can occupy one section at a time. Constraint programming that deals with problems defined within the finite set of possible values of each variable is the main technology used for solving mathematical formulation problems through the search trees. Fig. 2 shows an example of four feasible solutions to clarify the stages of obtaining these solutions using the search tree for the DFS algorithm, where each solution is shown by three subgraphs that start with discovering the nodes of the search tree to find the solution. The search tree uses coloured nodes to express the node types. For example, the red nodes are the failures, the solutions are green, the blue nodes are the explored choice points, white are the nodes created internally and still unexplored, and the black nodes are pruned points that appear in the CP Optimiser [4].

Metaheuristic techniques such as Simulated Annealing and Tabu Search are integrated with CP to improve the CP’s solutions [1], [2], [3], [4]. The use of the Gantt chart has been proven as a useful tool to validate the solutions’ applicability and to evaluate the algorithms’ performance through the ACTSS Schedule Checker for Kalamia Mill. As shown in Fig. 3, the different numbers of trains are indicated by using different colours to satisfy the specific allotment for each siding during a day. The rail sections have been constructed on the vertical axis while the time of each trip had been shown on the horizontal axis. The red numbers on the graph show the number of delivered empty bins and green numbers show the number of collected full bins at each siding.

## Figures and Tables

**Table 1:** Kalamia Mill’s sidings.

**Segment name**	**Siding index**	**Siding name**	**Capacity empty**	**Capacity full**	**Time from mill**	**Shunt time**
BRANDON/	1	H E A	242	242	6	15
GAINSFORD	2	KAL PLAINS	320	328	16	15
	3	BRANDON 1	272	272	18	15
	4	BRANDON 3	200	196	20	35
	5	BRANDON 4	328	328	0	10
	6	GAINSFORD 2	288	272	22	15
	7	GAINSFORD 4	320	320	26	20
CHIVERTON/	8	CHIVERTON 2	232	280	12	10
TOWN	9	CHIV TERMINUS	208	208	17	15
	10	LILLESMERE	264	264	8	10
	11	TOWN 3	440	440	12	20
	12	TOWN TERMINUS	264	240	15	30
MLINE/CENTRAL	13	MAIN LINE 1A	256	264	5	10
	14	MAIN LINE 1	112	136	6	10
	15	MAIN LINE 3	296	296	10	15
	16	MAIN LINE 4 A	200	200	14	20
	17	MAIN LINE 4B	192	192	14	20
	18	CENTRAL 1A	136	136	17	10
	19	CENTRAL 1	280	320	18	10
	20	CENTRAL 2	264	280	21	10
	21	CENTRAL 3	288	328	22	10
JARVISFIELD	22	JARVISFIELD 2A	208	264	15	10
	23	JARVISFIELD 2B	208	264	15	10
	24	JARVISFIELD 3	240	344	21	10
	25	JARVISFIELD 6	216	264	24	10
	26	J/FIELD TERM A	304	304	32	15
	27	J/FIELD TERM B	184	216	32	15
	28	JARVISFIELD 8A	224	248	26	15
	29	JARVISFIELD 8B	248	248	26	15
	30	JARVISFIELD 8C	208	208	26	15
NORHAM/IVANHOE	31	IVANHOE 2	376	376	16	10
	32	IVANHOE 3	257	273	16	10
	33	IVAN TERMINUS	240	240	21	15
	34	NORHAM 3	504	504	19	10
	35	NORHAM 4	240	256	25	10
	36	NORHAM DEPOT	240	240	27	10
RITA ISLAND	37	RITA ISLAND 4	248	312	30	10
	38	RITA ISLAND 6	232	272	35	10
	39	RITA ISLAND 7	248	248	36	10
	40	RITA ISLAND 9	104	144	42	10
	41	RITA ISLAND 10	200	224	46	10
	42	RITA ISLAND 12	200	224	50	10
	43	RITA ISLAND 15	184	184	55	10
	44	RITA ISLAND 16	248	256	58	10
	45	RITA ISLAND 17A	136	136	58	40
	46	RITA ISLAND 17B	160	160	40	40
MCDESME/AIRDALE	47	MCDESME 1	192	216	32	15
	48	2 MCDESME	206	206	35	10
	49	MCDESME 3A	344	352	45	15
	50	MCDESME 3B	344	352	45	15
	51	MCDESME 4	248	208	50	10
	52	MCDESME 5	208	240	55	10
	53	AIRDALE 1	256	224	60	10
	54	LAUNS	264	270	65	20
	55	AIRDALE 2	232	256	65	10
	56	AIRDALE 3	176	216	67	10
	57	AIRDALE 4	240	296	68	10
	58	AIRDALE 5	200	248	60	10
	59	AIRDALE 6	248	280	62	10
	60	AIRDALE 7	224	250	70	10
	61	SHEPPARDS RD	328	360	80	10
	62	BROWNS 1	224	264	80	10
LOOPS	63	BEACH LOOP	422	422	5	10
	64	AIRD LOOP	332	332	10	10
	65	MADDENS	558	558	13	10
	66	MCDESME 2	223	223	40	10
	67	BALLOON LOOP	429	429	5	10
BROWNS	68	BROWNS 1	224	264	80	10
	69	BROWNS 2	832	832	84	15
	70	BROWNS 3	248	272	112	20
	71	BROWNS 4	200	232	115	10
	72	BROWNS 5	320	328	95	20
	73	BROWNS 6	352	352	120	10
	74	BROWNS 7	848	848	100	15
	75	BROWNS 8	320	384	128	15
	76	MONA PARK 2	160	160	0	10
	77	MONA PARK 3	240	240	0	10
	78	MONA PARK 4	240	240	0	10

**Table 2:** Kalamia mill’s trains.

**Train order**	**Train name**	**Load empty**	**Load full**	**Speed empty**	**Speed full**	**Speed light**	**Average speed**
1	NORHAM	120	120	22	22	22	22
2	SELKIRK	120	120	22	22	22	22
3	BURDEKIN	120	120	22	22	22	22
4	STRATHALBYN	120	120	22	22	22	22
5	DELTA	120	100	20	18	20	20
6	AIRDMILLAN	100	80	20	18	20	20
7	CHIVERTON	100	72	20	18	20	20
8	KALAMIA	110	82	14	12	14	13.3
9	BOJACK	120	120	30	30	32	30.6
10	CARSTAIRS	110	90	28	22	30	26.6
11	NORTHCOATE	110	90	28	28	28	28
12	JARVISFIELD	120	120	34	34	34	34
13	RITA ISLAND	120	120	34	34	34	34
14	KILRIE	120	120	34	34	34	34

**Table 3:** Kalamia mill’s harvesters.

Group No	Harvester name	Enabled	Start time	Nom allot	Harvest rate
137	BUNDY	FALSE	5:00 AM	705	75
140	HAUGHTON/SUGAR	FALSE	6:00 AM	1140	75
206	DOWSON	TRUE	4:30 AM	986	90
208	DAVCO	FALSE	6:00 AM	0	140
212	ROCKS HARV	FALSE	6:00 AM	1381	1
216	KELLY	FALSE	6:00 AM	0	0.1
225	CHAPMAN	FALSE	6:00 AM	514	1
226	DENNIS	FALSE	6:00 AM	471	1
227	MCLEAN	FALSE	6:00 AM	651	1
229	GIDDY	FALSE	6:00 AM	801	1
231	VIERO	FALSE	6:00 AM	781	1
233	BUGEJA	FALSE	6:00 AM	760	62
234	NEWMAN	FALSE	6:00 AM	628	1
238	INVICTA 1	FALSE	6:00 AM	600	0.1
241	H.C.L.	FALSE	6:00 AM	1	75
242	DRAIN	FALSE	6:00 AM	664	1
245	SEXTON	FALSE	6:00 AM	692	1
246	MILLER	FALSE	6:00 AM	508	1
247	SPENCE	FALSE	6:00 AM	844	65
301	MUGUIRA	TRUE	4:30 AM	707	70
301	GALEA . P	FALSE	4:00 AM	1	70
302	T.F.D.	TRUE	7:00 AM	0	30
303	LAIDLOW	TRUE	3:30 AM	571	76
306	BONNANO.M.	TRUE	3:00 AM	645	75
310	TUFFIN. G.	TRUE	5:00 AM	494	70
311	BURKE.B.	FALSE	6:00 AM	0	1
313	SATORI.M.	TRUE	4:30 AM	550	65
320	NIELSEN.J.	TRUE	3:30 AM	593	76
321	SOUTHERN.J.	TRUE	3:30 AM	742	76
323	MCDONNELL	TRUE	6:00 AM	486	70
324	ARBOIT	TRUE	8:00 AM	243	26
330	BAPTY.S.	TRUE	6:30 AM	610	76
331	JONES	TRUE	7:00 AM	0	60
332	JONES. RYAN	TRUE	6:30 AM	730	70
333	COASTAL HARVESTING	TRUE	6:30 AM	697	90
341	OLSEN.M.	TRUE	6:30 AM	581	75
342	BONNANO BROS	TRUE	5:00 AM	612	70
352	MITCHELL.J.	FALSE	5:00 AM	445	80
353	BROMBAL	TRUE	2:30 AM	733	80
361	KELLY.J.	TRUE	4:30 AM	986	90
363	CARDILLO	TRUE	7:00 AM	69	26
364	SHERLOCK	TRUE	5:00 AM	404	70
373	SCUDERI.M.	TRUE	3:30 AM	931	85
380	MINUZZO. C	TRUE	4:30 AM	619	80
381	MALAPONTE	TRUE	5:00 AM	607	78
383	PIRRONE	TRUE	6:00 AM	437	20
391	QUAGLIATA.C.	TRUE	4:00 AM	809	90
393	BETTERIDGE S	TRUE	4:30 AM	625	70
394	DROVANDI	FALSE	7:00 AM	0	75
395	AHERN	TRUE	5:00 AM	563	75
398	iVORY 2	TRUE	6:00 AM	0	60
399	INKERMAN 1	FALSE	12:00 AM	0	0
400	SISL	FALSE	12:00 AM	600	1
401	INVOLATA	FALSE	12:00 AM	420	1

**Table 4:** Kalamia’s sectional rail network.

From Siding	To another Siding	Dist	From Siding	To another Siding	Dist
SHEPHERDS_JUNCT	BROWNS_1_	0.98	JN-38	BRANDON_3	0.94
BROWNS_1	BROWNS_2	1.06	BRANDON_3	BRANDON_4	1.6
BROWNS_2	BROWNS_3	1.16	JN-39	KAL_PLAINS	0.98
BROWNS_3	BROWNS_4	1.23	KAL_PLAINS	JN-38	1.82
BROWNS_4	BROWNS_5	2.54	JN-40	JN-39	1.59
BROWNS_5	BROWNS_6	0.24	JN-35	JN-40	0.48
BROWNS_6	BROWNS_7_	2.77	JN-40	H_E_A	0.32
BROWNS_7_	BROWNS_8	2.71	JN-41	GAINSFORD_2	0.27
SHEPHERDS_JUNCTI	SHEPPARDS_RD	3.23	JN-41	GAINSFORD_4	2.03
MONA_PARK_2	JN-15	0.74	JN-39	JN-41	4.22
JN-15	MONA_PARK_4	0.78	JN-35	CHIVERTON_2	1.68
JN-15	MONA_PARK_3	0.36	CHIVERTON_2	CHIV_TERMINUS	2.4
BROWNS_8	MONA_PARK_2	9.33	MAIN_LINE_4_A	MAIN_LINE_4B	0.01
LAUNS_POINTS	LAUNS	1.48	LAUNS_POINTS	AIRDALE_2	0.07
JN-21	RITA_ISLAND_17B	0.17	AIRDALE_2	AIRDALE_3	0.33
JN-21	RITA_ISLAND_17A	0.22	AIRDALE_3	AIRDALE_4	1.21
JN-22	RITA_ISLAND_15	0.31	AIRDALE_4	AIRDALE_5	1.48
JN-23	RITA_ISLAND_7	0.22	AIRDALE_5	AIRDALE_6	2.57
IVANHOE_POINTS	IVANHOE_2	1.2	AIRDALE_6	AIRDALE_7	3.81
IVANHOE_2	IVANHOE_3	1.24	AIRDALE_7	SHEPHERDS_JUNCTI	2.89
JN-27	JARVISFIELD_8A	1.46	MCDESME_4	MCDESME_5	1.41
CREEK_POINTS	JARVISFIELD_2A	0.76	MCDESME_5	AIRDALE_1	2.97
JARVISFIELD_2A	JARVISFIELD_2B	0.14	AIRDALE_1	LAUNS_POINTS	1.1
JARVISFIELD_2B	JARVISFIELD_3	1.75	RITA_ISLAND_PTS	MCDESME_1	1.3
JARVISFIELD_3	JARVISFIELD_6	1.39	MCDESME_1	MCDESME_3A	2.92
JARVISFIELD_6	JN-27	0.73	MCDESME_3A	MCDESME_3B	0
JN-29	JARVISFIELD_8B	0.39	MCDESME_3B	MCDESME_4	0.95
JN-27	J/FIELD_TERM_B	1.5	JN-22	RITA_ISLAND_16	0.71
CENTRAL_PTS_J8	CENTRAL_1A	0.33	RITA_ISLAND_16	JN-21	2.61
CENTRAL_1A	CENTRAL_1	1.4	JN-23	RITA_ISLAND_9_	1.57
CENTRAL_1	CENTRAL_2	1.06	RITA_ISLAND_9_	RITA_ISLAND_10	1.13
CENTRAL_2	CENTRAL_3	1.01	RITA_ISLAND_10	RITA_ISLAND_12	2.49
JN-33	MAIN_LINE_4_A	0.32	RITA_ISLAND_12	JN-22	0.82
JN-33	CENTRAL_PTS_J8	0.13	RITA_ISLAND_PTS	RITA_ISLAND_6	1.43
TOWN_PTS_J2	MAIN_LINE_1A	1.11	RITA_ISLAND_6	JN-23	1.73
MAIN_LINE_1A	MAIN_LINE_1	0.44	IVANHOE_POINTS	NORHAM_3	0.19
MAIN_LINE_1	MAIN_LINE_3	2.19	NORHAM_3	NORHAM_4	1.22
MAIN_LINE_3	JN-33	1.07	NORHAM_4	NORHAM_DEPOT	1.94
Mill	TOWN_PTS_J2	0.71	NORHAM_DEPOT	RITA_ISLAND_4	0.68
Mill	JN-35	0	RITA_ISLAND_4	RITA_ISLAND_PTS	0.67
JN-37	TOWN_3	1.53	CREEK_POINTS	IVANHOE_POINTS	1.9
TOWN_PTS_J2	JN-37	0.66	CENTRAL_PTS_J8	CREEK_POINTS	2
JN-37	LILLESMERE	0.36	J/FIELD_TERM_B	J/FIELD_TERM_A	0.43
JN-38	BRANDON_1	0.19	JN-29	JARVISFIELD_8C	0.37
JARVISFIELD_8A	JN-29	0.15	IVANHOE_3	IVAN_TERMINUS	0.99
