# Supplementary material for: Experimental dataset for optimising the freight rail operations
Source: Data Brief. 2016 Sep 21;9:492–500. doi: 10.1016/j.dib.2016.09.015 (PMC5053071; doi:10.1016/j.dib.2016.09.015)
Supplement: Supplementary file 1 — Supplementary material [file mmc1.pdf]

Conflict of Interest

The authors declare there is no conflict of interest.

The correspondence Author:
Mahmoud Masoud 16/08/2016
Dr. Mahmoud Masoud
